# Randomized Controlled Trial of Mindfulness Meditation and Exercise for the Prevention of Acute Respiratory Infection: Possible Mechanisms of Action

**DOI:** 10.1155/2013/952716

**Published:** 2013-09-29

**Authors:** Aleksandra Zgierska, Chidi N. Obasi, Roger Brown, Tola Ewers, Daniel Muller, Michele Gassman, Shari Barlow, Bruce Barrett

**Affiliations:** ^1^Department of Family Medicine, University of Wisconsin-Madison, 1100 Delaplaine Ct., Madison, WI 53715, USA; ^2^Schools of Nursing, Medicine and Public Health-Design & Statistics Unit, 600 Highland Avenue, University of Wisconsin-Madison, P.O. Box 2544, Madison, WI 53792, USA; ^3^Department of Medicine-Rheumatology Division, University of Wisconsin-Madison, 1 S. Park Street, Room 301, Madison, WI 53715, USA

## Abstract

*Background*. A randomized trial suggests that meditation and exercise may prevent acute respiratory infection (ARI). This paper explores potential mediating mechanisms. *Methods*. Community-recruited adults were randomly assigned to three nonblinded arms: 8-week mindfulness-based stress reduction (*N* = 51), moderate-intensity exercise (*N* = 51), or wait-list control (*N* = 52). Primary outcomes were ARI illness burden (validated Wisconsin Upper Respiratory Symptom Survey). Potential mediators included self-reported psychophysical health and exercise intensity (baseline, 9 weeks, and 3 months). A Baron and Kenny approach-based mediational analysis model, adjusted for group status, age, and gender, evaluated the relationship between the primary outcome and a potential mediator using zero-inflated modeling and Sobel testing. *Results*. Of 154 randomized, 149 completed the trial (51, 47, and 51 in meditation, exercise, and control groups) and were analyzed (82% female, 94% Caucasian, 59.3 ± SD 6.6 years old). Mediational analyses suggested that improved mindfulness (Mindful Attention Awareness Scale) at 3 months may mediate intervention effects on ARI severity and duration (*P* < 0.05); 1 point increase in the mindfulness score corresponded to a shortened ARI duration by 7.2–9.6 hours. *Conclusions*. Meditation and exercise may decrease the ARI illness burden through increased mindfulness. These preliminary findings need confirmation, if confirmed, they would have important policy and clinical implications. This trial registration was Clinicaltrials.gov: NCT01057771.

## 1. Introduction

Acute respiratory infection (ARI), also known as a “common cold” or “cold,” is extremely common, often debilitating, and easily transmittable. It is one of the top 10 most expensive illnesses [1] and one of the leading causes for health care utilization and school and work absenteeism [2–4]. Despite its high prevalence and impact, available treatments for ARI are not very effective and usually limited to symptomatic care [5, 6]. Development of new therapies for ARI prevention and/or treatment could tremendously benefit both individuals and society at large. 

It is common wisdom that being in a state of “good health” is one of the few measures one can do in order to prevent “a bad cold.” Research corroborates this belief by documenting links between poor psychophysical health and worse ARI-related outcomes [7–9]. Recently, two health behaviors, exercise and mindfulness meditation (“meditation”), have been gaining support as both “healthy lifestyle” modalities and medically-recommended therapies for a variety of conditions. Regular exercise may not only protect from ARI, but also decrease the duration and severity of ARI illness [10–12]. Evidence shows that meditation training can modulate immune response, including an enhanced response to the flu vaccine [13], and it can be effective for a variety of mental health problems, such as stress and depression [13, 14] that are known correlates of ARI illness severity [7–9].

With scientific evidence and public interest aligned in the search for effective therapies for the common cold, especially using “holistic” healthy approaches, we designed and conducted a three-arm randomized controlled trial (RCT) evaluating efficacy of meditation and exercise interventions for ARI illness prevention and treatment during a single cold/flu season (*N* = 154) [14]. Findings of this trial have been consistent with the existing literature and suggested that 8 weeks of training in meditation (Mindfulness Based Stress Reduction) [15] or moderate-intensity exercise can improve ARI-related outcomes. In this RCT, during the 3 month follow-up period, 149 participants provided outcome data and were included in analysis. Among them, 44% experienced an ARI illness and reported 27 ARI episodes in the meditation, 26 episodes in the exercise, and 40 episodes in the waitlist observational control group. Both meditation and exercise groups reported shorter ARI duration than controls (257 and 241 versus 453 days, resp.; one-sided *P* = 0.03). Compared to control, the meditation group also reported a statistically significant reduction in the global severity of ARI illness (*P* = 0.004), while the exercise group did not (*P* = 0.16). Similarly, the ARI-related absenteeism was lower in meditation (16 days, *P* < 0.001) and only marginally so in exercise (32 days, one-sided *P* = 0.04) group compared to controls (67 days) [14]. Interestingly, an evaluation of the individual ARI symptoms showed that the potential advantage of training in meditation over exercise for reducing cold and flu illness was explained as much or more by reduced functional and quality of life impact rather than by lower severity of individual ARI symptoms [16].

The current study, based on a mediational analysis from the above RCT (*N* = 149), was focused on exploration of possible mechanisms underlying efficacy of meditation and exercise interventions on primary outcomes: ARI illness duration and global severity, with an *a priori* hypothesis that reduction in perceived stress and/or increase in mindfulness scores mediated intervention effects.

## 2. Materials and Methods 

Details of the rationale and methods of the primary study are described elsewhere [14]. What follows is a brief description of methods relevant to this paper.

### 2.1. Design

Participants were randomly allocated into one of the 3 parallel equal groups: (1) meditation, (2) exercise, or (3) waitlist observational control. The primary RCT aim was to determine whether training in either intervention could reduce ARI illness burden, as compared to control (findings are published elsewhere [14]). Secondary aims were to test whether the training in meditation or exercise could improve psychosocial and physical health indices, and explore whether changes in these health indicators could explain or “mediate” intervention effects on primary, ARI-related outcomes; the current paper focuses on these secondary aims. 

### 2.2. Study Arms

The mindfulness meditation intervention, Mindfulness Based Stress Reduction (MBSR), was led by trained, experienced instructors through the University of Wisconsin (UW) MBSR program [17]. The standardized 8 week MBSR training included weekly 2.5 hour group sessions, 45 minutes of daily at-home practice, and a one day retreat, and promoted continued lifelong meditation practice [17]. The conceptual premise of mindfulness training, corroborated by evidence, is rooted in the idea that increased awareness of present-moment experiences can lead to a healthier or “mindful” response to challenges as opposed to reactive or habitual response.

The exercise program was designed and led by experienced Exercise Physiology staff from the UW Health Sports Medicine Center. It was matched to the meditation program in meeting location, contact duration, time and type, and home practice requirements. The goal was to achieve moderate intensity sustained exercise (target exertion rating of 12–16 points on the 6–20 point Borg's Exertion scale [18]). Group sessions were divided into didactic instruction and exercise practice. For most participants, home exercise consisted of brisk walking or jogging.

Waitlist observational control group participants were eligible to receive meditation training or monetary equivalent at trial's end and were monitored and evaluated using the same methods as the intervention groups. 

### 2.3. Participants/Setting

Community-based participants were recruited from Madison, WI and vicinity. The protocol was approved by the UW Health Sciences Institutional Review Board (protocol no. 2009-0075). The trial was monitored by a Data and Safety Monitoring Committee. The American Heart Association guidelines [19] for safety of exercising were followed.

Inclusion criteria included: age ≥50 years, reported ≥2 colds in the last 12 months, or ≥1 cold on average per year, and a successful completion of a 2 week long run-in trial. Exclusion criteria included: previous training in or current practice of meditation; moderate exercising ≥ twice/week or vigorous exercising ≥ once/week; score <24 points on the Folstein minimental exam [20]; score >14 points on the Patient Health Questionnaire-9 depression screen [21]; and self-reported immunodeficiency, autoimmune or malignant disease, or allergic response to flu vaccine or eggs. 

### 2.4. Recruitment and Monitoring

**Figure 1:**
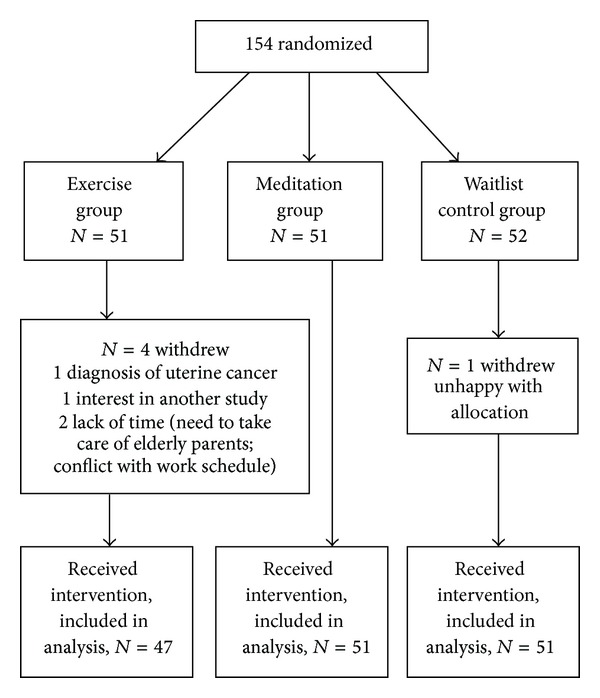
Participant Flow Diagram.

Potential participants, recruited primarily via media ads during the single cold/flu season (September 2009 and January 2010) were screened by phone (*N* = 833). Interested eligible adults then met with the study coordinator for enrollment procedures and entry into the 2 week long run-in trial (*N* = 204) whose completers were consented, enrolled, and randomized into the main trial (*N* = 154): 51 in the exercise, 51 in the meditation, and 52 in the waitlist control group. Participants were followed until study exit (May 2010); 149 participants provided outcome data (96.7% retention rate) and were included in analysis (Figure 1). 

### 2.5. Outcome Measures

All outcome measures were collected at baseline, 9 weeks (postintervention) and 3 months postentry. Starting at postintervention, participants were also monitored for ARI onset biweekly by telephone. Those who developed a cold were additionally asked to report their ARI symptoms daily.

The primary outcomes evaluated the ARI illness burden: severity and duration (no. days). The ARI illness presence was determined using the Jackson cold severity scale [22] and a 24-item version of the Wisconsin Upper Respiratory Symptom Survey (WURSS-24) [23–25] which adds items assessing headache, body aches, and fever to the well-validated WURSS-21 [26]. The WURSS-24 was filled out daily during the duration of each ARI episode. The illness severity during each ARI episode was assessed daily using the WURSS-24-scale [23–25]. For each subject, based on each ARI illness episode, global severity score (area under the curve, AUC) for all ARI illness days throughout the study was calculated, with the global score being the sum of scores for items 2 through 23 of the WURSS-24 questionnaire (first and last items were analyzed separately). ARI illness duration was assessed in minutes then converted to decimalized days. 

Health indicator outcomes included validated questionnaires inquiring about self-reported health. The 12-item Medical Outcomes Study Short Form (SF-12) measured overall health in physical and mental health domains [27]. The 10-item Perceived Stress Scale (PSS-10) evaluated severity of perceived stress; its score has been linked to ARI outcomes, including in influenza [8]. The Positive and Negative Affect Schedule (PANAS) [28] assessed positive and negative emotion levels, also known to be linked to ARI outcomes [29]. The six-item Life Orientation Test (LOT) assessed cognitive aspects of optimism [30]. The nine-item Positive Relationships with Others (PR) scale assessed perceived social support [31]. The 15-item one-factor Mindful Attention Awareness Scale (MAAS) measured the so-called degree of mindfulness [32]. The International Physical Activity Questionnaire (IPAQ) [33] measured the number of minutes and intensity of exercising. Spielberger's State Trait Anxiety Inventory (STAI) assessed severity of anxiety symptoms [34]. The Pittsburgh Sleep Quality Index (PSQI) [35] served as a measure of sleep quality. Details of other methods, not relevant to this analysis, can be found elsewhere [14, 36]. 

### 2.6. Randomization/Blinding

Using SAS software [37], 165 unique identification numbers were generated (balanced blocks of 3) and then concealed in sealed envelopes. The research coordinator opened consecutively-numbered envelopes after consent to determine allocation. Participants and assessors were not, but statistician was blinded.

### 2.7. Statistical Methods

SAS statistical program (version 9.2) was used for data analysis [37]; to evaluate possible mechanisms of intervention action, a complete data set was required (“per protocol” analysis). The sample size was determined *a priori* [14]. Two-sided *P* value ≤0.05 was considered statistically significant. Distributional data characteristics were assessed. Descriptive statistics were conducted with results presented as mean value ± standard deviation (SD) and 95% Confidence Intervals (CIs), unless otherwise specified.

The current paper is focused on evaluation of potential mechanisms that may underlie effects of the two interventions [14]. Based on the hypothesis that the score change in health indicators (“mediators”) may have mediated intervention effects on the ARI global severity (AUC) or illness duration (no. days) outcomes, mediational analyses examined the potential explanatory pathways using the statistical framework derived from the Baron and Kenny model [38], and modified by Krull and MacKinnon [39] and coauthor Brown [40]. Inclusion of a given health indicator into the mediational analysis model was based on (a) theoretical considerations, with the literature suggesting that improved health indicators, primarily the reduction in perceived stress (PSS scores) and/or increase in the degree of mindfulness (MAAS scores) could be “active ingredients” (“mediators”) underlying treatment efficacy and (b) results of prior analyses [14] showing that some, but not all, health indicators changed their scores over time within the group or when comparing active groups versus the control group. Scores of 7 health indicators, including the PSS and MAAS, that exhibited a statistically significant change [14], were included in modeling as potential mediators; four health indicators (PANAS-Negative, STAI, PSQI and SF-12 physical health) did not change and were, therefore, not included [14]. 

**Figure 2:**
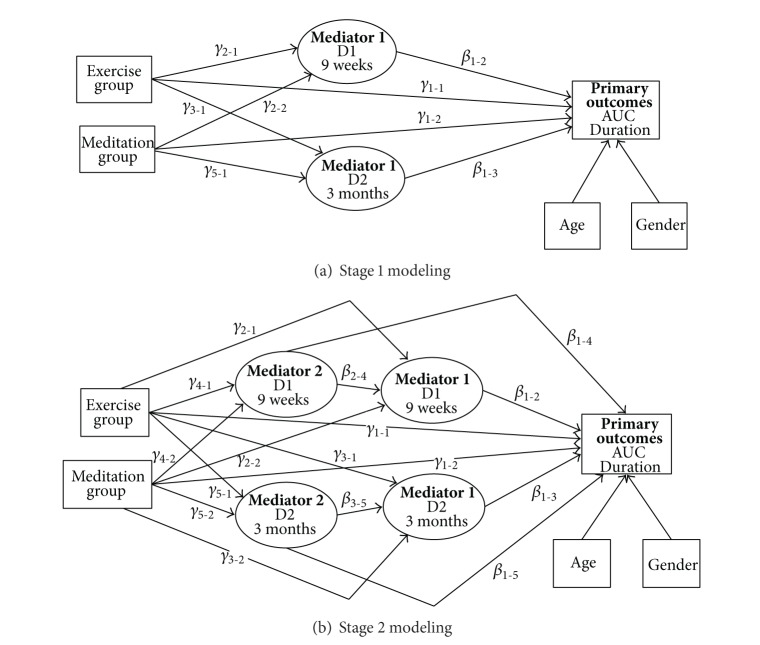
Mediational analysis models: the relationship between the “dependent variable” (primary outcomes: acute respiratory infection (ARI) severity and duration), “independent variables” (group status) and possible “mediators” (health indicators); separate models were created for each health indicator. Stage 1 models illustrate single-mediation, while Stage 2 models illustrate compound-mediation (change in the mindfulness score as Mediator 1, and change in the health indicator score as Mediator 2). *Abbreviations*/*Explanations*: AUC: area under the curve global ARI severity; “Mediator” is a proposed mediating variable; D1 refers to the “mediator” score change at 9 week, and D2 to the “mediator” score change at 3 month followup; *γ*1 pathways represent a direct relationship between group status and the primary outcome; *γ*2 through *γ*5 pathways represent relationships between group status and the potential mediator; *β*1 pathways represent relationships between the potential mediator and the primary outcome; and *β*2 and *β*3 pathways represent relationships between the two potential mediators.

Two types of mediational models, Stage 1 and Stage 2, were developed, differing in how the MAAS score was approached (Figure 2). The literature supports using the MAAS as either an outcome measure or health indicator (Stage 1 modeling) or as a “surrogate measure” that can reflect the intensity of mindfulness practice with the premise being that the more intense practice the larger increase in the MAAS scores (Stage 2 modeling) [41]. Each model evaluated a relationship between the ARI illness global severity or duration “dependent variable” and a health indicator that could mediate the intervention effects. Stage 1 models (Figure 2(a)) included the following: (i) the proposed mediating variable (its score change at 9 week [D1] or 3 month [D2] followup), (ii) group status (“independent variable,” expressed as two “dummy variables”: exercise versus control as Group 1, and meditation versus control as Group 2), (iii) age, and (iv) gender (Male = 1, Female = 0). Stage 2 models (Figure 2(b)) contained all Stage 1 variables and, in addition, included the change in MAAS scores at D1 and D2 (compound mediation structure). 

Because most participants did not report any ARI illness, the mediational analyses used a zero-inflated model (ZIM) approach [42, 43] to control for potential confounders and “adjust” for those who did not report any cold (“no-cold” participants, *N* = 83). Modeling data with a high number of “zeroes,” while ignoring such zero-inflation, could result in biased parameter estimates. The most common approach to modeling such distributions is to assume a logistic regression model for the “zero/nonzero" values of the outcome (i.e., “at least one cold episode” versus “no cold” participants) and a censored distribution for the model [44]. Because the zeros are accounted for in the logistic portion of the model, the variable portion can reflect, more accurately, the nonzero distribution. We used a censored-inflated regression model that estimated two equations: one for the continuous measures of ARI illness severity or duration and one binary (logistic regression). The continuous model was for participants who were above the censoring point (those who reported at least one cold); data distribution of the global ARI illness severity (AUC) was skewed and the Box Cox transformation normalized it. The binary model was contrasting participants who were above versus below the censoring point. As an outcome, each of Stage 1 and 2 models evaluated both (i) predictors of the ARI severity or duration among those who reported at least 1 cold and (ii) predictors of getting a cold. Mplus Version 6.12 [45] was used to construct the zero-inflated censored models. 

Once relationships between individual variables are established, the next step is to interpret these relationships in light of whether other variables may either influence or explain it; this latter case, of how other variables could explain the various relationships found in the studied causal pathways has been termed “mediation” [38] which was the focus of the current study. In general, mediation can be assessed by evaluating the products of the sequential pathways (Figure 2). Stage 1 or single-variable mediation models (Figure 2(a)) considered three separate relationships between (A) group status and primary outcomes (*γ*1 direct pathways); (B) group status and the potential mediator (*γ*2 and *γ*3 pathways), and (C) the potential mediator and primary outcomes (*β*1 pathways). To consider the presence of mediation, all three relationships, when considered individually, need to be statistically significant. Stage 2 or compound-variable mediation models (Figure 2(b)) accounted for the possible influence of two mediators: change in the MAAS score and change in the health indicator score. These models evaluated the relationships between (A) the group status and primary outcomes (*γ*1 direct pathways), (B) group status and the potential mediators (*γ*2 through *γ*5 pathways), (C) the potential mediators and primary outcomes (*β*1 pathways), and, in addition, (D) the two potential mediators (*β*2 and *β*3 pathways). The model suggests complete mediation when a direct *γ*1 pathway “loses” statistical significance after adjustment for the influence of a potential mediator or mediators (product of the *γ*2-5 pathways and *β* pathways). When the presence of significant direct influence of a given variable on the primary outcomes was suggested by Stage 1 and Stage 2 models (*P* < 0.05), the Sobel testing [46, 47] was used to further assess mediation. Health indicator could be considered a “true” mediator only if the results of Stage 1 or 2 modeling were corroborated by the statistically significant results of the Sobel test. The Sobel test involves computing the ratio of the product of the mediated effects along with an estimate of its standard error. A significant *P* value (*P* < 0.05) for the product ratio is used to support the hypothesis of mediation.

## 3. Results

Details on participant study flow, baseline characteristics, and primary outcome analysis findings were published elsewhere [14]. Briefly, participants (*N* = 149; Figure 1) were on average 59 (±6.6) years old, with 82% women, 94% Caucasian, 93% nonsmokers, 60% reporting college or postgraduate degrees, and 55% reporting annual household income ≥$50,000. Baseline measures were similar across the three groups.

Stage 1 modeling (Table 1) evaluated effects of individual health indicators (their change over time) on ARI-related outcomes when adjusted for age, gender, and group status (single mediation). It showed that change in the mindfulness score at 3 months (MAAS-D2) emerged as a potential mediator of beneficial intervention effects on both ARI illness global severity and duration (*P* < 0.05 for both *β* and *γ* pathways). Change in the optimism score was also identified as a possible mediator of intervention effects on ARI illness severity, suggesting that those with larger increases in optimism at 9 weeks reported more severe symptoms of ARI illness (LOT-D1, *P* < 0.05 for both *β* and *γ* pathways). However, this relationship trended in the opposite direction at 3 months, when those who endorsed more optimism were less likely to report severe ARI illness (LOT-D2, *P* = 0.06 for *β* pathway). Although changes in perceived stress (PSS-D2) and mental health (SF-12M-D2) scores also correlated to the ARI duration (*P* < 0.05 for *β* pathways), they did not appear to mediate intervention effects (*P* ≥ 0.05 for *γ*2 pathways). 

Stage 2 models (Table 2) assessed for the presence of compound mediation, by evaluating effects of individual health indicators on ARI-related outcomes when adjusted for age, gender, group status (as in Stage 1 modeling), and, additionally, for the change in MAAS scores. Stage 2 modeling indicated that change in the mindfulness score at 3 months (MAAS-D2) could be a potential mediator of intervention effects (*P* < 0.05 for both *β* and *γ* pathways) on the ARI illness severity (optimism, positive emotion, social support, and mental health models) as well as duration (exercise intensity, optimism, and social support models; and marginal significance in the mental health model, *P* = 0.056 for *β*13 pathway). In addition to mindfulness, change in exercise intensity also emerged as a possible mediator in the Stage 2 modeling for ARI illness severity, with increasing exercising at 9 weeks (IPAQ-D1) positively related to the increased cold severity (parameter estimate 0.3, *P* < 0.05 for *β*14 pathway). Although change in mindfulness score at 9 weeks (MAAS-D1, exercise model for ARI illness severity) and mental health score at 3 months (mental health model for ARI duration) correlated to the ARI illness severity and duration (*P* < 0.05 for *β* pathways), they were not likely mediators (*P* ≥ 0.05 for *γ* pathways). 

Overall, Stages 1 and 2 modeling indicated that an increase in the mindfulness score at 3 months (MAAS-D2) correlated to the decrease in ARI illness severity (*P* < 0.05; coefficient range from 333.1 to 484.5) and the reduction in ARI duration (*P* < 0.05). Across the models where MAAS-D2 appeared to be a potential mediator, each 1 point increase in the MAAS score corresponded, on average, to a shortened ARI illness duration by 7.2–9.6 hours (coefficient 0.3–0.4). Mediational analysis also showed a positive association between age and cold duration (but not severity) in several models (*P* < 0.05 for *γ*13 pathway): Stage 1 model for perceived stress (Table 1), and Stage 2 models for optimism, social support (Table 2), and perceived stress (details not presented), with estimated coefficient, on average, of 0.03 suggesting that with increase in age by 1 year, the cold duration lengthens by approximately 0.6 hours. Only one model showed a significant gender influence: in the Stage 2 exercise model for ARI illness severity, men were less likely than women to report severe ARI illness (*P* < 0.05 for *γ*14 pathway).

In addition to the investigation of potential predictors of the ARI illness severity and duration, the logistic portion of mediational analysis evaluated predictors of “catching a cold” versus not. Compared to those who reported at least one ARI episode (*N* = 66, “cold group”), the “no-cold” participants tended to be older (*P* < 0.05 in Stage 1 exercise and mindfulness models for ARI severity, and the perceived stress model for ARI duration; and in Stage 2 optimism, social support, and mental health models for ARI severity), with coefficient 0.2 in all but one model (Stage 1 exercise for ARI duration model estimated coefficient = 0.05). In addition, the no-cold participants were more likely to be in the exercise rather than the meditation group (*P* < 0.05; Stages 1 and 2 optimism models for the ARI duration; Stage 2 perceived stress model for the ARI severity; and Stage 2 positive emotion and mental health models for the ARI duration), with *β* estimate for exercise group of 0.9–2.6. When assessing the potential effects of health indicators, the no-cold versus cold participants only marginally differed in one of the health indicators: optimism score changed at 9 weeks (Stage 2 model for ARI duration, *P* = 0.046, *β* estimate 0.07) suggesting that the no-cold participants had a tendency to be more optimistic at the 9 week followup. Gender status reached significance in one model (Stage 2 perceived stress model for ARI severity; *P* < 0.05, *β* estimate −3.1) suggesting that women were more likely than men to report a cold.

Health indicators that, as based on Stages 1 and 2 modeling, appeared to be potential mediators, were then additionally examined using the Sobel analysis. The Sobel test, evaluating indirect mediational pathways, confirmed that change in the mindfulness score at 3 months can potentially explain intervention effects on the ARI illness severity (Table 2, *P* < 0.05 for MAAS-D2 in Stage 2 positive emotion and social support models). Change in exercising at 9 (IPAQ-D1) weeks was not corroborated by the Sobel testing to be a potential mediator of intervention effects on cold severity (Table 2).

## 4. Discussion

The primary goal of this mediational analysis was to explore potential mechanisms of action underlying efficacy of mindfulness meditation and moderate intensity exercise interventions, as compared to waitlist observational control, on ARI illness outcomes. Overall, Stage 1 and Stage 2 mediational analyses consistently indicated that improved mindfulness score, especially at the 3 month followup (MAAS-D2), may mediate intervention effects on ARI severity and duration (*P* < 0.05; direct mediational effects) and may, at least partially, mediate the relationship between ARI illness burden and the change in optimism, social support, mental health, and positive emotion scores. On average, each 1 point increase in the MAAS-D2 score corresponded to a shortened cold duration by 7.2–9.6 hours. In two Stage 2 models, the Sobel testing confirmed that mindfulness score change may mediate the beneficial intervention effects on ARI illness burden (*P* < 0.05). Although in several other models (both Stage 1 and Stage 2) the Sobel testing did not corroborate the presence of statistically significant indirect mediational effects of mindfulness (*P* ≥ 0.05), this may represent a “sensitivity issue” related to the small sample size since only 44% of participants reported having a cold during the study. Such interpretation is supported by the consistency of findings across the models, and the values of 95% confidence intervals for the Sobel test coefficient trending toward “zero” in multiple models.

The literature supports the hypothesis that improved mindfulness may be “the active ingredient” underlying intervention effects. An uncontrolled trial of MBSR (*N* = 121) found that self-reported time spent on meditation practice positively correlated to the improved mindfulness score (the Five Facet Mindfulness Questionnaire, FFMQ) which, in turn, mediated the relationship between meditation practice and improvements in psychological wellbeing and perceived stress at 8 weeks [48]. Participants in that study who meditated 31–35 minutes per day on average also reported a decrease (large effect size, Cohen's *d* = 0.9) in the medical symptom severity, consistent with our findings that increased mindfulness scores may mediate decreased ARI illness burden. An 8 week long RCT (*N* = 57) of community adults with increased distress, noted that the MBSR group, compared to waitlist controls, increased their mindfulness MAAS score which was identified as a potential mediator of intervention effects on perceived stress and quality of life outcomes [49]. Another RCT (*N* = 76), comparing effects of MBSR against a waitlist control for anxiety disorders, noted that change in the mindfulness score (FFMQ) was a potential mediator of improved anxiety outcomes [50]. Cross-sectional studies of chronic pain patients also suggested a strong link between mindfulness and health-related quality of life outcomes [51, 52].

Although change in the score of several other health indicators, such as optimism, perceived stress, and mental health status also emerged as possible mediators, these findings lacked the kind of consistent pattern that was apparent for mindfulness. Interestingly, exercise intensity did not seem to play a substantial role in Stage 1 modeling, but appeared to correlate to cold severity when adjusted for mindfulness (*P* < 0.05, Stage 2 modeling), with improvement in the MAAS score at least partially explaining effects of exercise on ARI outcomes. In general, change in the MAAS score was identified as the primary mediator underlying intervention efficacy in both exercise and mindfulness groups. This was not an expected result. It may be an incidental finding; however, another possible explanation is that changes in mindfulness may interact with or support the effects of exercise. Mindfulness scores rose (*P* < 0.05) in both intervention groups at 3 months [14], thus, suggesting that exercising may contribute to improved mindfulness which, in turn, can be a driving force behind the positive change in cold outcomes. This hypothesis may be supported by the fact that, while both meditation and exercise groups experienced fewer ARI illness days than controls, only the meditation group displayed a statistically significant reduction in overall ARI illness severity [14]. This interpretation does not speak against exercise, which is proven to support health, but, rather, highlights one of the possible mechanisms through which exercise exerts benefits.

Consistent with the existing literature [53–55], mediational analysis also showed that older individuals with ARI illness tended to experience longer cold duration (Stage 1 model for perceived stress and Stage 2 models for optimism, social support, and perceived stress (details of the latter analysis not presented)) and women were more likely than men to report more severe illness (Stage 2 exercise model). 

Logistic regression portion of mediational analysis evaluated factors that could differentiate between those who developed a cold versus those who did not. It indicated that older age, male gender, and being in the exercise group were the predictors of “no cold” status, findings which were also noted by others [55]. The “no-cold” participants were also more optimistic at 9 weeks, a finding consistent with the existing literature suggesting that optimism and other positive emotions may protect against infectious respiratory illness [9, 29].

Strengths and limitations of this RCT were described in detail elsewhere [14]. Of note, this was the first trial to evaluate effects of meditation and exercise on ARI illness outcomes in the settings of a comparative effectiveness RCT. The sample size may have been too small to draw firm conclusions from mediational analyses. Inability to blind participants and intervention instructors to the study arm could have introduced bias. We did not track meditation practice minutes during the study and so evaluation of “dose-response” relationship between meditation training and outcomes was not possible. There is diversity of opinion among researchers how best to evaluate the change in “mindfulness” resulting from a mindfulness training [56]; choosing a different questionnaire than the MAAS could have yielded different findings. Generalizability of results may also be limited by the fact that our sample was relatively healthy. At baseline, our participants reported less perceived stress (mean PSS-10 score: 11.9) and a higher degree of mindfulness (mean MAAS score: 4.6) than is usual [41, 57]. Over time, these scores further increased in the two experimental groups but remained unchanged in the control group. It is unclear who may benefit most from these interventions. It is possible that sicker, more stressed, and less mindful people could have gained more (“more room” for improvement). But the opposite may also be true, with early evidence suggesting that, compared to those with lower level of pretreatment mindfulness, individuals with higher pretreatment levels may experience larger increases in mindfulness and larger benefits from a mindfulness intervention [58]. In such a case, mindfulness “booster sessions” or even “retreatment” could facilitate additional gains. Due to the exploratory nature of this study, we elected to use unadjusted *P* values in our mediational models; although this approach allows for a more liberal investigation of possible mediational pathways which can be beneficial in exploratory investigations, it also accepts more “alpha error;” these results should be interpreted with caution. Future research, utilizing a larger sample size of less-healthy participants and controlling for dose-response effects of both meditation and exercise practices, can help corroborate the main findings and clarify mechanisms underlying efficacy. 

## 5. Conclusions

This randomized trial suggests that positive effects of meditation and exercise on ARI illness burden may be explained by improved mindfulness scores over time. Given that apart from hand washing and exposure avoidance, no ARI-prevention strategies have before been proven, these findings, while tentative, are especially noteworthy. Future studies with a larger sample size are needed to confirm these promising preliminary findings. If improved mindfulness is the “key ingredient” for ARI illness prevention and treatment, it would have important implications for health care practice and policy. 

## Figures and Tables

**Table 1:** Stage 1 mediational analysis models for ARI illness global severity and duration: main findings (*N* = 149).

Health indicator	Graphic symbol	Variables	Estimated coefficient	S.E.	*P* value	Sobel test single mediation coefficient (95% CI)
Stage 1 models for ARI illness severity (AUC): potential mediators						

Optimism (LOT) score	*β*12	(A) LOT-D1^∗1^	120.8	50.5	0.017*	
	*β*13	LOT-D2	−110.6	58.8	0.060	
	*γ*11	Group1	−232.4	377.3	0.538	
	*γ*12	Group2*	−471.7	192.4	0.014*	
		Age	12.8	55.4	0.818	
		Sex	11.1	347.6	0.975	
		(B) LOT-D1^1^ ON:				
	*γ*21	Group1*	1.6	0.5	0.001*	*γ*21-*β*12 = 193.2 (−2.0, 388.4)
	*γ*22	Group2	0.5	0.4	0.246	N/A

Mindfulness (MAAS) score	*β*12	(A) MAAS-D1	−269.4	230.0	0.241	
	*β*13	MAAS-D2^∗1^	−368.4	151.9	0.015*	
	*γ*11	Group1	365.7	300.2	0.223	
	*γ*12	Group2	−266.4	162.9	0.102	
		Age	12.1	16.8	0.473	
		Sex	47.3	203.9	0.817	
		(B) MAAS-D2^1^ ON:				
	*γ*21	Group1*	0.3	0.1	0.024*	*γ*21-*β*13 = −99.5 (−217.7, 18.7)
	*γ*22	Group2*	0.3	0.1	0.009*	*γ*22-*β*13 = − 117.9 (−246.7, 10.9)

Stage 1 models for ARI illness duration (no. days): potential mediators						

Perceived stress (PSS) score	*β*12	(A) PSS-D1	0.01	0.02	0.778	
	*β*13	PSS-D2*	0.06	0.03	0.023*	
	*γ*11	Group1	0.1	0.2	0.524	
	*γ*12	Group2	−0.3	0.2	0.141	
		Age*	0.03	0.01	0.033*
		Sex	0.06	0.2	0.793
		(B) PSS-D2 ON:				
	*γ*21	Group1	−1.6	0.9	0.081	N/A
	*γ*22	Group2	−1.7	1.0	0.093	N/A

Mental health (SF12M) score	*β*12	(A) SF12M-D1	−0.01	0.006	0.085	
	*β*13	SF12M-D2*	−0.01	0.002	<0.001*	
	*γ*11	Group1	0.03	0.2	0.864	
	*γ*12	Group2	−0.4	0.2	0.072	
		Age	0.02	0.01	0.134	
		Sex	0.006	0.2	0.972	
		(B) SF12M-D2 ON:				
	*γ*21	Group1	−1.3	6.5	0.842	N/A
	*γ*22	Group2	4.4	5.5	0.425	N/A

Mindfulness (MAAS) score	*β*12	(A) MAAS-D1	0.02	0.2	0.945	
	*β*13	MAAS-D2^∗1^	−0.4	0.2	0.037*	
	*γ*11	Group1	0.1	0.2	0.553	
	*γ*12	Group2	−0.3	0.3	0.172	
		Age	0.03	0.01	0.052	
		Sex	0.2	0.2	0.374	
		(B) MAAS-D2^1^ ON:				
	*γ*21	Group1*	0.3	0.1	0.024*	*γ*21-*β*13 = − 0.1 (−0.2, 0.02)
	*γ*22	Group2*	0.1	0.1	0.009*	*γ*22-*β*13 = − 0.1 (−0.3, 0.02)

Variables included: health indicator score change at D1 (9 weeks), and D2 (3 months), group status, age, and gender. Results presented only for the models that suggested presence of potential mediation. Sobel testing of indirect effects was conducted only for the identified potential mediators.

^1^Potential mediator (*P* < 0.05 for both *β* and *γ* pathways).

*P < 0.05.

**Table 2:** Stage 2 mediational analysis models for Acute Respiratory Infection (ARI) illness global severity and duration: main findings (*N* = 149).

Health indicator	Graphic symbol	Variables	Estimated coefficient	S.E.	*P* value	Sobel test single mediation coefficient (95% CI)	Sobel test compound mediation coefficient (95% CI)
Stage 2 models for ARI illness severity (AUC): potential mediators							

Exercise (IPAQ) METS	*β*12	(A) MAAS-D1*	−580.0	201.6	0.004*		
	*β*13	MAAS-D2	−132.2	162.5	0.416		
	*β*14	IPAQ-D1^∗1^	0.3	0.1	0.021*		
	*β*15	IPAQ-D2	−0.005	0.07	0.942		
	*γ*11	Group1	−174.4	237.6	0.463		
	*γ*12	Group2*	−344.6	164.0	0.036*		
		Age	29.2	23.0	0.204		
		Sex	−6.11	0.001	0.001		
		(B) MAAS-D1 ON:					
	*β*24	IPAQ-D1	<0.001	<0.001	0.259	N/A	N/A
	*γ*21	Group1	−0.009	0.101	0.928	N/A	N/A
	*γ*22	Group2	0.141	0.106	0.181	N/A	N/A
		IPAQ-D1^1^ ON:					
	*γ*41	Group1*	997.2	286.5	0.001*	*γ*41-*β*14 = 259.2 (−5.48, 524.04)	*γ*41-*β*24-*β*12 = −578.2 (−1813, 657.1)
	*γ*42	Group2	−186.4	266.0	0.483	N/A	N/A

Optimism (LOT) score	*β*12	(A) MAAS-D1	−305.9	207.9	0.141		
	*β*13	MAAS-D2^∗1^	−333.1	157.7	0.035*		
	*β*14	LOT-D1	51.4	49.2	0.296		
	*β*15	LOT-D2	−32.7	35.1	0.352		
	*γ*11	Group1	291.0	267.2	0.276		
	*γ*12	Group2	−304.7	165.7	0.066		
		Age	12.5	14.3	0.381		
		Sex	39.4	209.2	0.851		
		(B) MAAS-D2^1^ ON:					
	*β*35	LOT-D2*	0.1	0.02	0.013*	*β*35-*β*13 = −18.0 (−39.2,3.2)	N/A
	*γ*31	Group1*	0.2	0.1	0.045*	*γ*31-*β*13 = −76.6 (−180.0,26.8)	N/A
	*γ*32	Group2*	0.3	0.1	0.008*	*γ*32-*β*13 = −102.9 (−224.8,19.0)	N/A

Positive emotion (PANAS-P) score	*β*12	(A) MAAS-D1	−183.0	195.2	0.349		
	*β*13	MAAS-D2^∗1^	−484.5	163.0	0.003*	
	*β*14	PANAS-P-D1	−18.1	33.7	0.590	
	*β*15	PANAS-P-D2	246.6	39.5	0.238		
	*γ*11	Group1	482.8	278.6	0.083		
	*γ*12	Group2	−236.0	165.5	0.154		
		Age	3.4	22.2	0.878		
		Sex	127.0	202.7	0.531		
		(B) MAAS-D2^1^ ON:					
	*β*35	PANAS-P-D2*	0.03	0.009	0.001*	*β*35-*β*13 = −14.3 (−27.4,−1.7)*	N/A
	*γ*42	Group1*	0.24	0.12	0.038*	*γ*31-*β*13 = −115.8 (−249.0,17.5)	N/A
	*γ*52	Group2*	0.27	0.12	0.022*	*γ*32-*β*13 = − 130.3 (−271.5, 10.9)	N/A

Social support (PR) score	*β*12	(A) MAAS-D1	−320.8	207.8	0.122		
	*β*13	MAAS-D2^∗1^	−437.4	169.8	0.010*		
	*β*14	PR-D1	−0.89	8.8	0.920		
	*β*15	PR-D2	28.8	17.5	0.1		
	*γ*11	Group1	387.0	278.0	0.164		
	*γ*12	Group2	−246.3	165.4	0.137		
		Age	11.3	13.6	0.405		
		Sex	34.1	203.5	0.867		
		(B) MAAS-D2^1^ ON:					
	*β*35	Ryff-D2*	0.04	0.01	<0.001*	*β*35-*β*13 = −18.8 (−36.4,−1.2)*	N/A
	*γ*31	Group1*	0.2	0.1	0.028*	*γ*31-*β*13 = −104.9 (−227.9,18.0)	N/A
	*γ*32	Group2*	0.3	0.1	0.017*	*γ*32-*β*13 = − 118.9 (−252.1, 14.2)	N/A

Mental health (SF12M) score	*β*12	(A) MAAS-D1	−259.5	230.6	0.260		
	*β*13	MAAS-D2^∗1^	−355.2	153.5	0.021*		
	*β*14	SF12M-D1	0.9	8.8	0.919		
	*β*15	SF12M-D2	−2.08	2.7	0.446		
	*γ*11	Group1	396.6	316.4	0.210		
	*γ*12	Group2	−253.3	174.6	0.147		
		Age	9.4	230.6	0.658		
		Sex	27.3	153.4	0.896		
		(B) MAAS-D2^1^ ON:					
	*β*35	SF12M-D2*	0.005	0.002	0.012*	*β*35-*β*13 = −1.8 (−3.8,0.3)	N/A
	*γ*31	Group1*	0.3	0.1	0.016*	*γ*31-*β*13 = −96.6 (−201.0,16.8)	N/A
	*γ*32	Group2*	0.3	0.1	0.015*	*γ*32-*β*13 = −105.8 (−229.2, 17.5)	N/A

Stage 2 models for ARI Illness Duration (no. days): potential mediators							

Exercise (IPAQ) METS	*β*12	(A) MAAS-D1	0.07	0.3	0.788		
	*β*13	MAAS-D2^∗1^	−0.4	0.2	0.046*		
	*β*14	IPAQ-D1	<0.001	<0.001	0.591		
	*β*15	IPAQ-D2	<0.001	<0.001	0.127		
	*γ*11	Group1	0.2	0.2	0.311		
	*γ*12	Group2	−0.3	0.2	0.164		
		Age	0.02	0.01	0.207		
		Sex	0.2	0.2	0.451		
		(B) MAAS-D2^1 ^ON:					
	*β*35	IPAQ-D2	<0.001	<0.001	0.17	N/A	N/A
	*γ*31	Group1*	0.2	0.2	0.044*	*γ*31-*β*13 = −0.1 (−0.2,0.03)	N/A
	*γ*32	Group2	0.3	0.1	0.01*	*γ*32-*β*13 = − 0.2 (−0.3, 0.03)	N/A

Optimism (LOT) score	*β*12	(A) MAAS-D1	0.01	0.2	0.964		
	*β*13	MAAS-D2^∗1^	−0.4	0.2	0.047*		
	*β*14	LOT-D1	−0.01	0.04	0.893		
	*β*15	LOT-D2	−0.01	0.05	0.807		
	*γ*11	Group1	0.1	0.2	0.522		
	*γ*12	Group2	−0.3	0.3	0.186		
		Age*	−0.4	0.01	0.047*		
		Sex	0.2	0.2	0.376		
		(B) MAAS-D2^1^ ON:					
	*β*35	LOT-D2*	0.05	0.02	0.013*	*β*35-*β*13 = −0.02 (−0.04,0.005)	N/A
	*γ*31	Group1*	0.2	0.1	0.045*	*γ*31-*β*13 = −0.08 (−0.2,0.03)	N/A
	*γ*32	Group2*	0.3	0.1	0.008*	*γ*32-*β*13 = − 0.1 (−0.2, 0.03)	N/A

Social support (PR) score	*β*12	(A) MAAS-D1	−0.005	0.2	0.894		
	*β*13	MAAS-D2^∗1^	−0.4	0.2	0.041*		
	*β*14	PR-D1	−0.002	0.02	0.908		
	*β*15	PR-D2	0.01	0.02	0.495		
	*γ*11	Group1	0.1	0.2	0.523		
	*γ*12	Group2	−0.4	0.2	0.15		
		Age*	0.03	0.01	0.044*		
		Sex	0.02	0.2	0.371		
		(B) MAAS-D2^1^ ON:					
	*β*35	PR-D2*	0.04	0.01	<0.001*	*β*35-*β*13 = −0.02 (−0.04,0.001)	N/A
	*γ*31	Group1*	0.2	0.1	0.028*	*γ*31-*β*13 = −0.1 (−0.2,0.03)	N/A
	*γ*32	Group2*	0.3	0.1	0.017*	*γ*32-*β*13 = − 0.1 (−0.2, 0.03)	N/A

Mental health (SF12M) score	*β*12	(A) MAAS-D1	0.1	0.3	0.677		
	*β*13	MAAS-D2^2^	−0.3	0.2	0.056^2^		
	*β*14	SF12M-D1	−0.009	0.007	0.183		
	*β*15	SF12M-D2*	−0.006	0.002	0.007*		
	*γ*11	Group1	0.2	0.2	0.391		
	*γ*12	Group2	−0.3	0.3	0.228		
		Age	0.02	0.01	0.088		
		Sex	0.1	0.2	0.489		
		(B) MAAS-D2^2^ ON:					
	*β*35	SF12M-D2*	0.005	0.002	0.012*	*β*35-*β*13 = −0.001 (−0.04,0.004)	N/A
	*γ*31	Group1*	0.3	0.1	0.016*	*γ*31-*β*13 = −0.09 (−0.2,0.03)	N/A
	*γ*32	Group2*	0.3	0.1	0.015*	*γ*32-*β*13 = − 0.1 (−0.2, 0.03)	N/A
		SF12M-D2 ON:					
	*γ*42	Group1	−1.3	6.4	0.843	N/A	N/A
	*γ*52	Group2	4.3	5.4	0.426	N/A	N/A

Variables included: the MAAS score change at D1 (9 weeks) and D2 (3 months), health indicator score change at D1 or D2, group status, age, and gender. Results presented only for the models that suggested presence of potential mediation.

*P < 0.05.

^1^Potential mediator (*P* < 0.05 for both *β* and *γ* pathways).

^2^Trend toward being a potential mediator (*P* = 0.056 for *β*13, and *P* < 0.05 for *γ* pathways).
